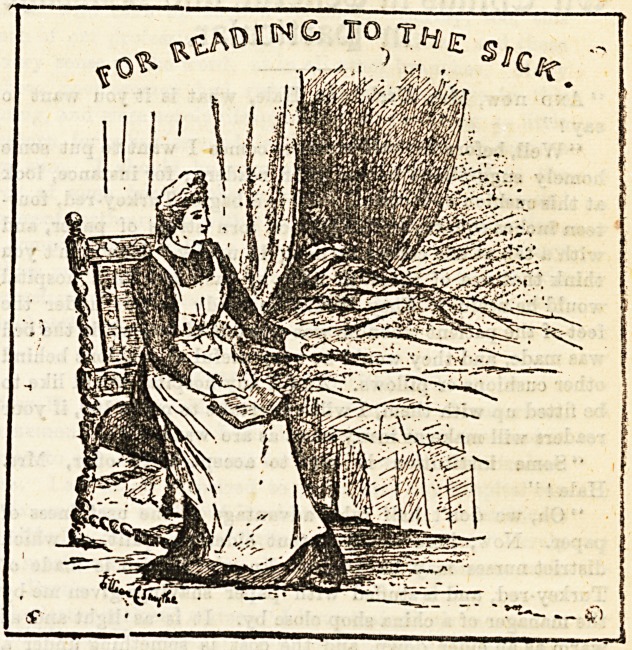# The Hospital Nursing Supplement

**Published:** 1891-10-10

**Authors:** 


### "The Hospital" Nursing Mirror.

Being the Extra Nursing Supplement of "The Hospital" Newspaper.

Contributions for this Supplement should be addressed to the Editor, The Hospital, 140, Strand, London, W.C., and should have the word "Nursing" plainly written in left-hand top corner of the envelope.

### En Passant.

**SHORT ITEMS.**—The Lady Superintendent of the Nurses' Co-operation requests us to state that the Committee have decided to enroll no more nurses this year; there are already 200 nurses on the books.—Miss Magee has succeeded Miss Wilson at Highgate.—The private nurses of University now receive an extra 5s. a week while they are at cases. Considering how low their salaries are, this is a step in the right direction.—There has been an epidemic at St. Thomas's, and six of the nurses are leaving to be married; we are glad to say none of them are marrying members of the staff.—A concert has been held on behalf of the Jarrow Nursing Guild, which consists of ladies holding the St. John's Ambulance Certificate.—Miss Sidné Reed of the Birkenhead Fever Hospital, has been asked to resign.—The Nurses of the City of London Lying-in Hospital have been practising fire drill.—Last week's *Queen* contained an account of the Sisters of East Grinstead.—Sister Anne Luithead has been presented with a dinner-service on leaving Silver Street; she and Mrs. Batty are returning to New York.

**THE HAMILTON ASSOCIATION.**—The sixth annual report of this Association tells of thirty-seven male nurses on the books, of whom, on May 30th, twenty-seven were employed as follows: Hospital work, seven; attending as masseur on private patient, one; attending as nurses on private patients, nineteen. The receipts for the year were only £20 short of £3,000, so that the Association is to be congratulated on its success. The rules have been modified, so let us hope that those male nurses who wrote and complained to us at one time are now satisfied. Fines have been inflicted on several nurses, and three men have been expelled during the year. In theory, the male nurse and the female doctor are excellent; in practice, we find that they are both regarded as a rule with distrust. "I cannot get the same sympathetic and careful nurses amongst men," complained a physician to whom we strongly recommended male nurses. "I cannot feel the same safety in the hands of a lady: she seemed so weak and helpless," said the patient who had tried a lady doctor.

**SIX MONTHS' CERTIFICATES.**—It is with the greatest surprise we have this week discovered that the Middlesex Hospital gives its paying pupils a certificate at the end of six months, stating that they are *qualified to discharge the duties of a trained nurse*. This is the power of the guinea with a vengeance. The duties of the lady pupils at the Middlesex are well known; they do not come on duty till 8.30, they have an hour for their dinner, and they are off duty for two hours in the afternoon. Yet it seems that they can in these few hours daily for six months learn what it takes the average hard-working probationer at least one year, and generally three to learn. Imagine a "lady" who has gone through these six months presenting herself at a private case, perhaps an infectious case, where she only has charge of the room and the patient; is she qualified to keep the room clean and tidy and nurse the patient? She has probably no notion of sweeping or dusting, she can have but little notion of nursing, and yet the authorities of the Middlesex Hospital have certified that she is "qualified to discharge the duties of a trained nurse." The Middlesex Hospital has a good reputation, let us hope it will maintain it by immediately removing this blot from its nursing system.

**LINCOLN NURSES.**—It has been decided to hold a bazaar in the Autumn of 1892 in aid of the Institution for Nurses at Lincoln. It is to be a big bazaar, for a big sum is needed. The institution supplies the town with seven district nurses and it supplies nurses at a low charge to those of the middle classes who cannot afford the usual fees. The result is a deficit of about £130 on the year's working. Miss Boucherett, Mrs. Sebthorp, and Miss Bromhead are getting up the bazaar; and Miss Bromhead as Lady Superintendent and Treasurer of the Institution, knowing well the wear and tear of fighting hopelessly against a deficit, is sure to throw herself heartily into the scheme. She has our very best wishes for a successful sale.

**MR. JAMES PAYNE ON NURSES.**—It seems we have wronged Mr. James Payne—he denies that he has ever, to his knowledge, made a joke upon any such subject as nursing. Will our readers then please take seriously that shilling shocker of Mr. Payne's we recommended to them eighteen months ago as an amusing bit of literature? Also, should that railway friend "£200 Reward" fall into their hands, will they please understand that the sketch of a nurse given therein must be taken as truth. We are afraid this will lower their estimation of Mr. Payne; but still, since he is so hurt at the imputation of humour, why we must e'en believe that Nurse A. B., who murdered three of her patients, was a personal acquaintance of his, and not a mere effort of his imagination. But we should like to give a quotation:—

"Do you remember the very stout gentleman, doctor—him with the appleplexy in Ward No. 2—at St. Barnabases?"

"446. Pleurisy. Left convalescent?" inquired I, from memory.

"The same, sir. I bled him to death, doctor, at his own house within the week. His friends paid me by the job, you see, and I was overanxious to get it over."

"Good Heavens!" cried I; "and to save yourself a little trouble, you committed, then, a cruel murder?"

"He went off like a lamb," cried the wretched creature apologetically. "But there's worse than that. I once gave a young gent four doses of laudanum in one, and you wouldn't a known when he was dead from when he slep'. But them was murders for all that, I know."

"They certainly were, miserable woman," cried I indignantly. "Have you anything yet more upon your mind?"

"Hush!" whispered she, pointing towards the door; "she's listening; they always does it, bless you—I knows 'em so well. Once—only once, as I'm a sinful woman—I smothered a sick man with his pillow; that was for his money; he would have died any way, because he had the lock-jaw. Now," added she, with a long-drawn sigh, and after a pause, "I feel somehow better and more comfortable-like, thanks to you, Sir."

The patient had sunk back from her sitting-posture, as if exhausted with this terrible narration; but I read in her yet anxious eyes that she had still something more to say. Presently she again broke silence, and this time the emphasis with which she spoke was mingled with a tone of gratitude. She desired to recompense me, I suppose, for my prompt attention and interest, and delivered herself of this advice instead of a fee:

"When *your* time comes, doctor, and your friends send for the nuss, *don't let them pay her by the job*."

Mr. Payne says he has many reasons for being grateful to the calling of nursing, it is an odd way to show it if the above must indeed be taken seriously.

### On Private Nursing.

The few words of counsel and advice to nurses which have already appeared have been addressed to those whose work lies in the wards of a public hospital, where the life is hard, the toil incessant, and the responsibilities at times almost overwhelming, so that patience and temper are liable to break down from the strain.

We now turn to the subject of private nursing. Here the duties are lighter, the responsibility lessened by the nearness of the patient's relations, and the nurse herself so protected by the rules laid down for her benefit, that she has fewer temptations to fight against, and those of a totally different character. Indeed, to the lay mind there is something fascinating in the idea of nursing in the homes of the wealthy, with their airy, spacious apartments and every appliance which money can command for comfort and convenience, so that there is little occasion and less excuse for any display of temper or discourtesy. A true lover of the profession would feel with Mark Tapley, "there was no credit in being jolly under the circumstances." Still, there is a small opening left for a certain amount of tyranny, such as keeping the anxious friends rigorously from the bedchamber, under pretext of the patient requiring perfect quiet, and vouchsafing no definite information to allay said relatives' fears. We knew one nurse who, with every social advantage, kept a poor patient in a state of abject dependence on her will (she was certainly very trying with fidgets and worries, but she was also very weak and in a critical condition, and knew it). It was known afterwards that the attendant had been crossed in love just at that time, which accounted for much, yet one of the first duties of a nurse is to put self on one side, and if she felt unequal to doing so, she ought to have relinquished the case, and not made the poor patient suffer.

Again, the nurse is quite right in a general way to insist on having her stipulated hours of rest, and a proper amount of nourishment at stated times, especially when the case is tedious and prolonged; but under certain circumstances, such as an unexpected and fatal conclusion of an illness, with its consequent inevitable confusion, or the sudden breakdown of a servant, would it not be graceful, nay, Christianlike, to waive her claim for the first attention in the household, and even condescend in some measure to wait on herself?

Then with some there is a sad "reversion to types," a tendency to say with the immortal Sairey, "I takes but little, but I takes it good," and "put it on the mankle shelf and let me help myself when I'm so dispoged," so that much friction ensues about the quantity, quality, and regularity of the meals. As we have said before, nurses are but women, and have rocks ahead in life on which, without care, they may split their little barque. If they take the Great Pilot on board, they will be able to avoid them, and steer safely through every shoal and sandbank without once running aground.

One very serious count remains, *i.e.*, nursing people of limited means in their own houses. Unfortunately they are no more exempt from sickness than the rich, who have every advantage; or the poor, who have the hospitals to fly to. Many dread to hear the fiat go forth, "This case requires a trained nurse," for they feel on the horns of a dilemma, either the chances of prolonging a valuable life or being cramped by the inevitable expense. They know their already overburdened drudge will find it the last ounce she can bear, and they fear the incomer will give herself airs at the scantiness of her surroundings and the quality of her food, though she might see, "an she would," that the rest of the household do not fare sumptuously.

Happily this middle class can be accommodated in some measure by the new arrangement for paying in the large hospitals, but where one can be taken in hundreds must languish without proper attendance. It is for these, dear friends, for whom I would ask your pity and consideration. Bring your full sympathy to bear on them, for if you in health feel the uncomfortableness of straitened circumstances what must it be to those who are suffering! You are doing Christ's work quite as much in waiting on them and soothing them as in giving your time and care to the poverty-stricken. He will appreciate your kindness to them as done unto Himself. Try and live in the atmosphere of His love, He will help you to choose the right path, though it be a narrow and stoney one. In the warmth of His love difficulties will vanish, duties become light, tempers softened, and on leaving you will be followed by the blessings of those who, at first dreading your approach, have learnt to see that the trained nurse is one of the greatest benefits of the nineteenth century.

### A Bed for a Sick Nurse.

A fortnight ago we made an appeal for twenty-seven subscribers of a guinea each to endow a bed for a sick nurse. We explained that we had asked nurses to subscribe to so many schemes lately, that we wanted this time to find twenty-seven nurse's friends to aid us. The very day we issued the appeal we received the following letter:

"Dear Mr. Editor,—What a charming idea is 'A Bed for a Sick Nurse,' and at dear old St. Leonards, too—that's nice. May we not ask each and every nurse to send you sixpence towards it? they will never miss that small sum, yet if every one of the 2,000 Pension Fund Nurses will do it what a nice little cheque you could hand over towards the bed. For which of us we know not, dear fellow nurses, may be the next to be called aside 'to rest awhile' listening to our Heavenly Father's 'Fear thou not, for I am with thee?' I enclose my sixpence and beg to remain, dear Sir, yours in deep gratitude, —One of the 'First Thousand.'"

A delightful letter, but once more we repeat that we are ashamed to ask nurses for more sixpences; we are not in the least ashamed to ask the many readers we know we have who are not nurses, for a guinea from each. But so far as the guinea goes, the only response has been from Miss Yorke, Lady Superintendent of the Macclesfield Infirmary, who writes:

"I have much pleasure in sending a guinea subscription towards endowing a bed for the use of nurses. The suggestion is a most admirable one; I think our best thanks are due to those friends who are so willing to care for nurses needing rest and change. The Committee of this Infirmary have requested our Secretary to send you a cheque for a guinea early next week. I hope the new departure made by the managers of the Brassey Home will soon be followed by other 'Homes' nearer our large manufacturing towns."

This pleasant note decides us to believe that our readers are holding back because they thought there would be a run of subscribers; but please remember we have now only five out of the thirty, and that we want twenty-five more as soon as possible. If these twenty-five are not forthcoming (which we cannot believe possible) then we shall avail ourselves of the suggestion in the first letter, and collect the money in sixpences, acknowledging with General Booth that it is the poor who are most generous. In connection with this appeal we have also received a letter from Miss Wood of the Children's Home, Albion Terrace, Ramsgate, who says she would receive a nurse for a month's change in return for a little assistance with needlework and taking the children for walks.

---

The imposition of a fine upon a German doctor for publishing the names of his patients in conjunction with the clinical accounts of their cases is a light punishment for an unpardonable breach of confidence.

### The Princess of Wales and the Nurses.

The following is the final list of names of nurse subscribers to the Screen Fund. So many nurses have been away for their holidays during the last two months, that we have had some difficulty in procuring a sufficient number of photographs. The number received does not reach the total which was published as the minimum required. In consideration of the disappointment which would be caused were the scheme to fall through, we have reduced the size of the screen, which we believe will in no way lessen the beauty of its appearance :—J. E. Murray, A. M. Rowson, G. B. Calpin, A. M. Brown, H. M. Freeman, G. A. Daniels, R. Turner, E. Sully, L. Gillham, T. Groves, M. Mitchell, C. Leakey, L. Taylor, H. Cooper, C. Clark, H. B. Legg, M. Parker, E. Bayliss, — Pitt, A. Smith, L. Wills, H. Hoing, E. Toms, S. Johnson, M. Carter. List of Extra Subscriptions from the nurses: M. Thomas, 10s. ; H. Bailey, 3s. 6d. ; G. Smith, 2s. ; M. A. Fraser, 2s. ; C. Dunn, 3s.; Showler, 2s. ; S. Sudgel, 3s.; S. Taylor, 3s. ; T. C. Pocock, 3s. ; A. E. Steer, 10s. 6d.

Errata.—E. S. Maur for E. L. Mann, Corbell for Corbett. The subscription of 2s. from Nurse A. Lee in our last list, should have been acknowledged as an additional contribution.

### Where to Go.

At St. George's Hall on October 18th, at 4 p.m., Sir James Crichton Browne will lecture on "Brain Rust." On October 25th Mr. Frank Kerslake will lecture on "Rabies and Hydrophobia," seats 1s. and 6d. At the Nurses' Club, 12, Buckingham Street, the opening lecture of the session will be given on Friday evening, October 16th, at 7.45, by Mr. Gustavus Hartridge, F.R.C.S., &c., Surgeon to the Royal Westminster Ophthalmic Hospital, Ophthalmic Surgeon to St. Bartholomew's Hospital, &c. Subject: "The Structures and Functions of the Eye." A few tickets to non-members sixpence each.

### Love Divine.

*Infirmary Hymn circulated by the Edinburgh Tract Society.*

O heavy heart, with sorrow bowed,
Behold the rainbow in the cloud !
Know for thy joy that in this spot
Where suffering is the common lot,
God loveth thee and loves thee well.

Though summer's hand thou canst not see
When laid on grass and flower and tree,
Be this the comfort where thou art,
Making glad summer in thy heart,
God loveth thee and loves thee well.

Though winds blow easterly and bold,
As sheep thou art within the fold.
No winter touches thee with frost,
For love the threshold once hath crossed :
God loveth thee and loves thee well.

The kindness that surroundeth thee,
The tender, patient ministry,
So gently shown, so freely given,
Confirm the truth from highest heaven—
God loveth thee and loves thee well.

If thou shouldst walk the world again,
Freed from the shackles of thy pain
And all the weariness that was,
God makes thee free and glad because
He loveth thee and loves thee well.

But if He bring thee where the sea
Of death lies, full of mystery,
Lean on His hand, it will not fail,
Christ died for thee, thou must prevail,
God loves in Christ and loves thee well.

### GOD'S PROMISE POINTED OUT.

A writer in *The Christian* has lately pointed out how much real joy and strength we lose for the battle of life, aye, and for death too, because we either are ignorant of God's promises, or forget them in the time of need. When we are lying sick and afflicted on the couch of anguish, seemingly about to part from all we hold most dear, and helpless to renew the struggle against the stream which is carrying us on we know not whither, the words "Yet the Lord thinketh upon me" should come as an assurance full of encouragement and strength. It matters little, though the tired brain cannot think for itself, there is One taking charge of us who will not let a sparrow fall to the ground without His knowlege. "The Lord thinketh upon me." Take hold of these words. "The Lord whose sphere of thought is so much larger than mine ; whose power of thought is so much stronger than mine ; whose quickness of discernment is so much keener than mine ; how many resources which never occurred to me lie open to His survey ! And it is a fact that He really thinks upon you. Is He not your Father, your Redeemer, your Saviour Can your heavenly Father forget "that you have need of all these things ?" Can any part of your affairs escape His attention, who numbers the hairs of your head ?

Having interposed to deliver you from sin and its consequences by the sacrifice of the Cross, will He withhold from you what little thought is all sufficient for your temporal well-being ? Designing to make you an heir of glory, will He suffer you to be overwhelmed with earthly calamities ? Take hold of the assurance, "The Lord thinketh upon me." It is as true for you as for the psalmist to whom it was first given, as true as that you exist ; for you personally and all your history are the outcome of His thought.

The fear had oppressed you that all your resources had failed, that no one cared much for you, or, if a few friends did care, they were powerless to help. And, lo ! the Lord is thinking upon you. The Lord in heaven, who beholdeth all the inhabitants of the earth, has His eye and His heart upon you individually. You are precious in His sight. He is thinking how this calamity, these straits, fears, and forebodings, may promote your higher life ; may break entanglements which bind you to earth ; may compel you to draw nearer to Him, and enter more fully into the light of His countenance. And He is thinking, too, how far this trial may go, when and how it should end, and what new experience shall succeed. "Darkness may endure for a night, but joy cometh in the morning." Take hold and keep hold of the promises.

### On Things in General, and Massage in Particular.

"And now, Mrs. Creighton Hale, what is it you want to say?"

"Well, before the cold weather comes I want to put some homely suggestions before your readers; for instance, look at this cushion or footstool. It is a bag of Turkey-red, fourteen inches square, stuffed full of torn strips of paper, and with a tab at one corner to hang it up by. Now don't you think that one of these to hang by each bed in a hospital would be useful? They would be ready to put under the feet of the patient when he was got out of bed while the bed was made, and they would be very useful as cushions behind other cushions or pillows. And if any hospital would like to be fitted up with them, I will undertake to make 100, if your readers will make as many more as are wanted."

"Some institution is sure to accept that offer, Mrs. Hale!"

"Oh, we don't half take advantage of the usefulness of paper. Now, here is my patent eiderette quilt, of which district nurses may be glad to know. It also is made of Turkey-red, and is stuffed with paper shavings given me by the manager of a china shop close by. It is as light and as warm as an eider down, and the cost is something under a shilling. Then think how easily the paper can be burnt, and the cover disinfected, if needful."

"Well, really, Mrs. Hale, one wonders why poor people shiver when such a simple remedy is at hand."

"Then again, straw mattresses can be made for under two shillings; the bag should be kept at the district nursing home, and restuffed for each case to which it is lent. Or the minister's wife, or whoever looks after the comforts the poor need when they are sick, could keep these mattresses. And old corks neatly arranged in Turkey-red sewn into pipes, make such useful footstools for poor old rheumatic people, whose feet should never rest on the floor. And when nurses appeal for 'old linen' they make such a mistake; they should say 'white rag,' for many people think cotton is no use in nursing, whereas old cotton underclothing torn up is most useful for the numerous cases of 'bad legs,' which exist everywhere."

"Were you ever a district nurse, Mrs. Hale?"

"Oh no! but I go amongst the poor. But I have one very particular suggestion for hospitals and institutions; that they should each keep a trestle standing four feet high, and supplied with a hard mattress for massage cases. It is so very difficult to mass a patient on an ordinary low bed; one's back aches so with the stooping. The trestle would cost very little; I have one upstairs you can see when my pupils have gone."

"Have you many pupils just now?"

"Yes; I get so many pupils who have been taught elsewhere after a fashion—such a fashion, if you would believe me! One pupil told me her doctor had taught her on a pillow. Another wrote to me to ask if she could be taught by correspondence. It is extraordinary the notion some people have of massage! Look at this advertisement, for instance, of a woman who describes herself as 'an ablutionist, beautifier, manicure, and masseuse.' That looks as though massage were a sort of adjunct to the toilet of ladies who are too grand to clean their own nails. Then I hate to see single lessons in massage advertised, for, really, the full course is absolutely necessary, and I know that women who only take a few odd lessons in massage go and set up as masseuses."

"How long do you consider it takes to learn massage?"

"That depends; the movements can be learnt in a fortnight, if the whole time be devoted to the work. My pupils attend daily, and each pupil has to be 'dummy' at least once. This teaches them better than any amount of showing, and is much more merciful than practising on patients perhaps suffering from rheumatic gout, as is done in some hospitals. Some pupils I have to refuse to teach; if they stayed here months they would never learn. One woman with a bent finger came to me, but I told her at once she would never make a masseuse. Another came who was hopelessly clumsy with her left hand; she also had to be dismissed."

"No, it is theoretical as well; and at present I am engaged

"Is your teaching all practical, then, Mrs. Hale?"

in writing a book on massage. See, here are some of the illustrations. It will be very fully illustrated—in fact, I mean to make it as complete in every way I can."

"Then it will be an expensive book, I suppose?"

"About 6s., I expect; it can't be less if I illustrate every movement on every limb, as I want to."

"Have you a class going on just now, Mrs. Hale?"

"Yes; would you like to come up?"

"Very much indeed." We went up to a large bedroom where the trestle bed occupied a centre position; on the bed, wrapped in a blanket, was the pupil acting "dummy," one beautiful white arm uncovered while it was being practised on. In another part of the room a pupil with short hair had been pressed into the service to be "dummy" for the massage of the head and face. To the uninitiated it looked as though she were having her eyes gouged out, but "dummy" seemed quite happy under it. "Yes, it is the best method of learning, to be practised on," said one pupil, "but it makes you so hungry; even rubbing makes you terribly hungry, and it is just time for lunch now.' After which hint we promptly took our leave.

### Everybody's Opinion.

*[Correspondence on all subjects is invited, but we cannot in any way be responsible for the opinions expressed by our correspondents. No communications can be entertained if the name and address of the correspondent is not given, or unless one side of the paper only be written on.]*

### OBJECTIONABLE ADVERTISEMENTS.

A "Mother of Ten" writes: There is an almost grandmotherly legislature that watches over the morals of the British public, and tells them what they may or may not read in the way of light literature. There is an all-beneficent County Council ever ready to secure peace of mind to the British matron by the prompt removal of the counterfeit presentments of too lightly clad music-hall divinities. Cannot either of these "powers that be" do something to suppress, or at least divert into a proper channel, such advertisements as the enclosed? It is not taken from a paper intended for the perusal of ladies only, but from a fashionable magazine for general reading, and the "Genuine Prize Competition," &c., was read out in all innocence by one of my young daughters, a girl just budding into womanhood. Imagine her shame and confusion when she got past the first line! Not a day passes but I take out of my letter-box pamphlets and circulars quite unfit for the reading of my children and servants, the people into whose hands such things usually fall; and I really do think as I eschew spicy French novels, and don't go to music-halls, I might be allowed an immunity from "objectionable advertisements."

### NURSES AS PATIENTS.

"S." writes: Having recently had to undergo a serious operation, I was myself obliged to be a patient for two weeks in one of our general hospitals in London. Thus I felt deeply the truth of most of the remarks written in The Hospital, September 19th, "A Few Words to Nurses." Although a nurse of 16 years' experience and 44 years of age, I am of a sensitive nature, therefore suffered by the noisy, brusque, and hurrying way in which the rules were constantly performed by nurses years younger than myself—rules that need not have been disagreeable to any right-minded person, if differently carried out. I used to frequently think those nurses would never be kept a day as a "private" nurse in many families, if they spoke to the patient as they used to speak to their hospital patients. Words that were not objectionable, only made so by the manner in which they were spoken. I do not believe any woman, gentle or simple, can make a real "sympathetic" nurse unless she has a pure love for the work. Nothing can be more irksome, I should think, than nursing if merely followed as a means of making money. May I also take this opportunity to state how much comfort I derived during my illness from my sick pay allowance from the Pension Fund? When I joined the fund three years ago I never dreamt of ever having to undergo an operation, but it was a great relief to me, and helped my recovery, to know that I was not dependent on my friends.

### AN APPEAL FOR DOLLS.

MISS ANN PIGOTT, Matron Superintendent, Royal Infirmary, Preston, Lancashire, writes: A competition and exhibition of dolls will be held in this town in December. The object is to supply all the children in the Royal Infirmary and Orphanage with a doll at Christmas. There will be five classes, two prizes in each, 10s. and 5s. The dolls for competition must be dressed with only hand-work and, the clothes must not be washed. Will any nurses help by sending a doll dressed in the uniform of her hospital or home? In the event of any one not caring to compete, still if they will send any kind of doll and put a ticket on it "not for competition," it need not then be dressed by the sender. In this way nurses can get a convalescent patient to help. Also nurses will sometimes find patients willing to dress a doll; in that case the patient can compete. As almost 150 are required to carry out the plan fully it is hoped everyone will do a little and give pleasure to the little folk by allowing of each to possess a doll of her own. It is requested that all dolls will be sent to me by December 1st.

### MONTHLY NURSES.

A "MONTHLY NURSE" writes: May I ask you to insert this letter in reference to the remarks of "S." on "Three Months' Certificates"? Like "S.," I trained in a London lying-in hospital in 1889, and having had every experience in nursing, went for two months' training and certificate. In applying for a vacancy I was asked to furnish references, which I supplied, and in due time received a letter from the Matron, saying I was accepted, and stating that I was expected to "enter on my duties" on December 9th, at three p.m. Arriving at the hospital in good time, I was (after paying my training-fee of £11 6s.) shown into a very dismal underground apartment called a "sitting-room," and a "sitting-room" it proved to be in a very literal sense, for there I sat, together with several others as unfortunate as myself, for *three weeks*, by the scanty light in which I had to learn all that was necessary (or was supposed to be so in this institution) for the care and skill required at the hands of nurses for women in the most critical (as I have since found it to be) period of their lives. Whilst there I had the charge of four cases; and this, with a few lectures, was all the training I received for my fee of £11 6s. A certificate was given to me, and I learnt that a similar certificate was given to every woman who paid the training-fee, and who did not "misbehave" herself during her stay there, irrespective of whether she was fitted for such work or not. The women I met there were chiefly servants (though this was no discredit to them), and many of them had no idea whatever of the meaning of "taking notes," nor that innate refinement of feeling and manners so necessary in all classes of cases of this kind. Surely the "system" of training "monthly nurses" needs reformation, and a higher standard of education demanded of those who take up such a vocation. Surely, too, this branch of our profession needs educated "ladies," and these in every sense of the word, as in all other branches. Many of these poor women had risked all their savings in this training, and were sorely disappointed at receiving so little in return for what seemed (and no doubt was to them) a large sum. I should be very sorry to find myself at the mercy of some few of the so-called "certificated nurses" who went forth armed with their eight-weeks' certification, and comparatively nothing more, from that hospital, many of them never having been in a sick room before and utterly unconscious of the numerous sore tests to which their very limited skill and knowledge would be put.

### SIX MONTHS' CERTIFICATES.

MISS H. MORANT, Matron of the National Sanatorium, Bournemouth, writes: I enclose a letter I have received in answer to my advertisement in THE HOSPITAL for a trained nurse. I am much surprised to learn that any hospital can in six months send out probationers with a certificate "qualified to discharge the duties of a trained nurse." Surely there must be some mistake. It took me three years to train at St. Bart's.

[The letter enclosed states: "I have trained six months at the Middlesex Hospital, W., May to October, 1890, and have certified as trained nurse. The certificate states that I have passed through the medical and surgical wards for the period mentioned, and that I am qualified to discharge the duties of a trained nurse."]

### Examination Questions.

WITH the coming of the long evenings we once more commence our monthly competitions, which we hope probationers and district nurses, and all nurses debarred from many lectures or classes, find useful. This winter we mean to introduce one or two new features, and, first of all, we offer a book on nursing as a prize for industry; that is, for the nurse who answers regularly for six months in the most correct manner. For October we offer a pocket medical dictionary for the best drawing of a skeleton, all the bones being numbered, and their names written against similar numbers below. The skeleton must not be more than seven inches tall and three inches broad; it must be drawn in pen and ink on a piece of cardboard. It may be copied, but not traced. The name and address of the nurse must be written on the back of the card. All drawings must be addressed, "Nursing," THE HOSPITAL, 140, Strand, London, W.C., and must reach this office by October 24th. Only nurses are eligible for this competition.

### Notes and Queries.

### Answers.

(48) Put 2 lbs. loaf sugar in quart jug and fill to the brim with water. Boil quarter of an hour. Mix together 1 oz. powdered citric acid, with 50 drops essence of lemon, and stir into the syrup when cool and bottle. A very delicious drink, and only slightly acid, the amount of sugar counteracting the acidity and rendering it smooth. One tablespoon to half tumbler of water, or according to taste.—*M. S.*

(51) It ought not to be against a nurse to have acted as matron of a refuge; I was matron of an orphanage before I came here.—*T. W.*

*R. S.*—Playfair's "Midwifery" is the standard book; it is published at £1 8s., but you could probably get a second-hand copy at Kimpton's, 82, High Holborn, for less.

*Argyle Lodge, Bournemouth.*—We should be glad to have the names and addresses of any nurses who stayed at the above address during August or September.

*Nurse Edith.*—No post-office will receive letters except to a person's full name. You had better write to the Benevolent Branch of the Pension Fund, 8, King Street, Cheapside, London. We believe part of their scheme is to grant loans to nurses.

*Nurse B.*—Dr. Dundas Grant, 17, Finsbury-square, E.C.

*Nurse E.*—A monthly nurse has no distinct duties while waiting save to see that all arrangements are made for the event, and help with these, particularly with any needlework that may want doing.

*Mrs. L. P.*—We will notice your suggestion next week. Surely, if you let your doctor know he would sometimes be most glad of your help with poor cases?

*Gipsy.*—No, it still exists, and at the old address, but 130 members resigned last year.

### Kitty's Find.

### HOME AGAIN.

"You must come with me, Allie." Kitty had recovered herself, and was conscious of the rapt interest developed by the hoppers close by in the encounter between herself and the hapless waif whom she had recognised as pretty Allie Beecher, the village blacksmith's only child, who had shot up simultaneously with herself, the vicar's daughter, into early womanhood. But their paths had suddenly diverged, and now they met in a hop-garden, the one girl's pure, noble face telling she had found out for herself that

"Life is real! life is earnest!"

the other—but who is there cannot picture the face of a woman with a past?

"Come!" went on Kitty, gently, and already the sun had sunk behind the distant dark woods, the signal for the hoppers to leave off picking, and betake themselves to the huge shed where they supped and passed the night.

"Don't touch me!" cried a harsh voice, as Kitty's soft little hand tried to seize one of the thin brown ones pressing the apron to Allie's face; "I'm not fit, Miss Kitty, not fit."

"Allie! Is it so?" Kitty's clear, wistful eyes looked up into the shrouded face.

"It isn't as you think, Miss Kitty." The apron was flung desperately aside, and Allie held out her left hand, on which gleamed a wedding-ring. "But—but, oh, what a life I've lived since I left the old home!" and there was a catch in the speaker's breath.

"Come with me," again said Kitty, quietly; "we must find a lodging somewhere for the night." The pickers had all departed, and the hop-gardens were empty.

"Miss Kitty, I said true when I told you not to touch me for I wasn't fit. Listen! I've been in gaol—for thieving. He, the man I married, deserted me, to starve or steal, so I stole—again and again. Oh, go away, Miss Kitty! The sight of you brings it all up again—the old life, the old home!"

"And the old people, Allie? The father and mother who go mourning all their days, grieving for their lost girl."

Allie shivered, and her hands went out as if to push off some sight her eyes dreaded to behold. Allie had been the prettiest girl in the village, or "a rare tidy lass" as her people loved to call her. So pretty, that one of the gentleman pupils at the farm flirted with her, and Allie imagined meant to marry her. But the lad had no such thought; to him the episode was but a summer idyll, to be forgotten when winter came. The girl, hurled out of her fool's paradise, was maddened by the dispelling of her dream when her lover deserted her. She quailed before the prospect of her companions' jeers and sneers, she who had held her head so high. Finally, she ran away, to hide her sorrow—some did not hesitate to say in death. But foolish Allie, though stung to the heart, was not crazed enough for that. After a reckless marriage with a stranger, who, in his turn, deserted her, she went slipping and sliding down until, as she told Kitty, she stole to live.

"And I'm as good as a murderess, for I've killed the girl you were so good to in the old days, Miss Kitty!"

"No, no!" said Kitty earnestly. "There's not much of the old Allie left, but you've not killed her outright. My dear, you must let me nurse back to life and health—you know, it's my profession—that sick, despairing soul of yours, as well as your ailing body. And when some of the old Allie's looks come back, we shall go home to the village, you and I."

"Miss Kitty!" The poor, torn, tattered thing stood still to gaze with wonder-wide eyes, at the speaker. "You to be seen with me!"

Kitty smiled slowly. She was remembering One who came to seek and save the lost sheep; who was not ashamed to be seen in the company of sinners.

Side-by-side Kitty and her "find" picked in the days that followed, the former fighting valiantly with the black despair that clutched Allie in its tight grasp. But without knowing it, the miserable woman was already straighter; it had set her up on her feet, among the crew of hoppers to have such as Kitty calling her friend. Before the end of the week Allie also was, by Kitty's endeavours, more suitably "clothed," and, perhaps as a sequence, almost in her "right mind," that is, she began dimly to understand that life's stains might be washed away.

"Well!" Kitty gave a tired but happy sigh at the end of her real good time. "I actually thought I was taking my own way in striking out this new idea for a holiday, but it seems it's nothing of the sort. I've simply been sent to find a straying sheep, and now I must lead it home."

It was the old story of the Prodigal's return when Kitty and her "find" went back to the village. Not a word of reproach was cast at the trembling sinner. Tenderly the old folk gathered to their hearts their lost and found sheep, and by their faithful love Allie's wounds will be healed. And Nurse Katherine, as she moves about in the wards, smiles happily, remembering her holiday-spell, when she worked so hard in the hop-gardens, both among the bines, and at her trade of healing.

### Amusements and Relaxation.

### SPECIAL NOTICE TO CORRESPONDENTS.

**Fourth Quarterly Word Competition commenced October 3rd, 1891.**

Competitors can enter for *all* quarterly competitions, but no competitor can take more than *one first prize* or *two* prizes of any kind during the year.

Proper names, abbreviations, foreign words, words of less than four letters, and repetitions are barred; plurals, and past and present participles of verbs, are allowed. Nuttall's *Standard* dictionary only to be used.

The word for dissection for this, the SECOND week of the quarter, being

"CONTEST."

| Names. | Oct. 1st. | Totals. | Names. | Oct. 1st. | Totals. |
|---|---|---|---|---|---|
| Paignton | — | — | Christie | — | 44 |
| Psyche | 144 | 635 | Dulcamara | 123 | 598 |
| Hope | — | 647 | Nurse J. S. | 131 | 604 |
| Lightowlers | 135 | 647 | Qu'appelle | 146 | 660 |
| Wizard | — | 179 | E. M. S. | — | 68 |
| Wyameris | — | 46 | Jenny Wren | 139 | 606 |
| Dove | — | 46 | Carpe-diem | — | 65 |
| Punch | — | 181 | Grannie | — | 36 |
| Ivanhoe | — | — | Nurse G. P. | 90 | 382 |
| Tinie | — | 93 | Goodnight | — | 122 |
| Agamemnon | 145 | 640 | Gamp | — | 100 |
| Nurse Ellen | — | 86 | Charity | — | 104 |

### Notice to Correspondents.

**Results of Third Quarterly Word Competition.**

*First Prize*, 15s., is awarded to Qu'appelle (Miss Greaves, Barncote, Reigate).

*Second Prize*, 10s., is awarded to Lightowlers (Miss Chadwick, 2, Canning Street, Liverpool).

*Third Prize*, 5s., is awarded to Agamemnon (Mr. H. Hull, Queen's Road, Weybridge).

All letters referring to this page which do not arrive at **140, Strand, London, W.C.**, by the *first post on Thursdays*, and are not addressed PRIZE EDITOR, will in future be disqualified and disregarded.

N.B.—Each paper must be signed by the author with his or her real name and address. A *nom de plume* may be added if the writer does not desire to be referred to by us by his real name. In the case of all prize-winners, however, the real name and address will be published.

## Figures and Tables

**Figure f1:**